# Identification of Inhibitors of Biological Interactions Involving Intrinsically Disordered Proteins

**DOI:** 10.3390/ijms16047394

**Published:** 2015-04-02

**Authors:** Daniela Marasco, Pasqualina Liana Scognamiglio

**Affiliations:** Department of Pharmacy, Centro Interuniversitario di Ricerca sui Peptidi Bioattivi (CIRPEB), University of Naples “Federico II”, DFM-Scarl, 80134 Naples, Italy; E-Mail: liana.sco@gmail.com

**Keywords:** flexible protein regions, peptides, human diseases

## Abstract

Protein–protein interactions involving disordered partners have unique features and represent prominent targets in drug discovery processes. Intrinsically Disordered Proteins (IDPs) are involved in cellular regulation, signaling and control: they bind to multiple partners and these high-specificity/low-affinity interactions play crucial roles in many human diseases. Disordered regions, terminal tails and flexible linkers are particularly abundant in DNA-binding proteins and play crucial roles in the affinity and specificity of DNA recognizing processes. Protein complexes involving IDPs are short-lived and typically involve short amino acid stretches bearing few “hot spots”, thus the identification of molecules able to modulate them can produce important lead compounds: in this *scenario* peptides and/or peptidomimetics, deriving from structure-based, combinatorial or protein dissection approaches, can play a key role as hit compounds. Here, we propose a panoramic review of the structural features of IDPs and how they regulate molecular recognition mechanisms focusing attention on recently reported drug-design strategies in the field of IDPs.

## 1. Structural Features and Models of PPI Hubs in Networks

Intrinsically disordered proteins (IDPs) are not endowed with stable tertiary structures but are involved in recognition processes with other biomolecules (including proteins): during the formation of complexes the disordered protein regions simultaneously undergo to folding and binding events. These regions are usually specific motifs, named molecular recognition features (MoRFs), bearing short sequences able to transit from disordered to partially ordered configurations following a fly-casting mechanism [1]. In principle, a polypeptide can potentially follow three structural ways: nonfolding, folding and misfolding, and these two last routes are competitive and can cause aggregation/fibrillation and functional oligomerization of the ensemble of molecules [2]. A highly charged polypeptide with low overall hydrophobicity will not fold giving rise to an extended disordered region, while a balance of polar and hydrophobic residues will lead to a folded state. However, some changes in the amino acid sequence (point mutations) may favor the misfolding pathway for both the natively unfolded and folded protein regions [3]. IDPs exist as dynamic ensembles, “protein clouds”, in which the atom positions and backbone Ramachandran angles vary significantly over time with no specific equilibrium values and typically they undergo non-cooperative conformational changes. Statistical analysis shows that amino acid sequences encoding for ID regions are significantly different from those of ordered proteins on the basis of local amino acid composition, flexibility, hydropathy and charge [4,5]. Unlike globular proteins, disordered segments are rich in charged amino acids, deficient in hydrophobic residues, and have a low degree of complexity and can adopt collapsed structures whose degree of compaction can be modulated by charge interactions [6].

In the architecture of protein complex, IDPs usually provide a large interface due to their higher net charge and lower hydropathy in respect to structured proteins [7]; indeed, disorder makes them suitably flexible and malleable in order to adapt to different interfaces and to increase interaction surface areas, facilitating low-affinity/high-specificity binding [8]. Thus, disordered binding regions provide specific but transient interactions that enable IDPs to play central roles in signaling pathways [9,10] and Protein Protein Interactions’ (PPI) networks: these involve few proteins with many partners (called hub proteins or hubs) and many proteins with a few partners [11,12,13]. Several hub proteins are entirely disordered but capable of binding large number of partners while others contained both ordered and disordered regions and the most part of the interactions mapped to the disordered regions [12,14]. The most common use of disordered regions by hub proteins is to bind to multiple partners [13,15] even if there are several examples of linear interaction motifs with largely overlapping functional properties. This mode is focused on distilling a short consensus sequence pattern from proteins with a common interaction partner. These motifs reside in disordered regions and are considered to mediate the interaction roughly independent from the rest of the protein [16]. PPI networks are considered scale-free, with most of the proteins having only one or two connections but with relatively fewer hubs possessing tens, hundreds or more links: intrinsic disorder can serve as the structural basis for hub protein promiscuity. Indeed, IDPs can bind to structured hub proteins and flexible linkers between functional domains enable mechanisms that facilitate binding diversity [12].

## 2. IDPs and Diseases

The recent importance of IDPs and hybrid proteins, containing ordered and disordered regions (IDPRs), points out a key role for functional disorder in cell regulation and although they are normally tightly controlled, rigorous investigations of IDP functions and dysfunctions led to the recognition that they are prevalent among disease-related proteins and that many human diseases are based on the inability of a protein region to adopt its functional conformational state, leading to protein misfolding, loss of biological activity, gain of toxic function and/or protein aggregation [17,18]. Often the disruption of disorder by disease-associated mutations impairs interactions with corresponding partners [19]. Not casually intrinsic disorder is highly abundant among proteins associated with human diseases, giving rise to the D2 (disorder in disorders) concept: it states that when IDPs are mutated or in a changed environment they not only respond by misfolding but also by mis-recognition, as a consequence of the altered recognizing patterns, leading to diseases’ rising and onset [17]. Along with D2 theory, another proposed effect is related to the “molecular titration”: it is based on the impaired availability of IDPs entrapped by other proteins through non-functional interactions causing an imbalance in signaling pathways [20]. Since IDPRs can both form stable complexes or transient signaling interactions jumping quickly from bound/unbound and ordered/disordered states, an intriguing hypothesis is that disease’s mutations can cause an inverse transition from disorder to order. From an *in silico* study carried out by mutating proteins bearing disease mutations and then comparing the predicted disorder scores of wild-type and mutated proteins, it was found that disease mutations lead to predicted “disorder to order” transitions more frequently than polymorphisms not associated with diseases or neutral evolutionary substitutions. This suggests that transitions of disordered regions into folded states may play important roles in various diseases [21].

Uversky’s group was the most active in pointing out the involvement of IDPs in human diseases [22]. Indeed, by applying protein disordered region predictors (such as PONDR VL-XT [23]) to cancer associated proteins, they observed a significant enrichment of proteins with IDPRs among these proteins compared to other eukaryotic proteins. Examples of IDPRs cancer proteins include p53 [24], BRCA1 [25], EWS [26], HPV proteins [27] and PTEN [28]. IDPs also characterize human neurodegenerative diseases (as reported in Table 1): Parkinson’s disease, dementia with Lewy bodies, and Down’s syndrome involve the accumulation of α-synuclein protein that is able to assume a variety of conformations depending on the cellular environment [29] while Creutzfeldt-Jakob disease, scrapie, bovine spongiform encephalopathy are caused by prions, and ataxin (spinocerebellar ataxia) and Alzheimer’s disease (AD) by amyloid β and τ proteins [30]. The involvement of IDPs in pathogenesis of human diseases has been investigated in many computational/bioinformatics studies to evaluate the abundance of IDPs in various pathological conditions. Many algorithms based on solved or modeled protein structures combined with evolutionary conservation have been developed to predict the functional effect of mutations and to distinguish between damaging and benign mutations [31]. The analyses of genomic sequences revealed that protein disorder is prevalent and increases with evolutionary complexity [32]. Through disorder predictors [11,30], it was established that 79% of cancer-associated and 66% of cell-signaling proteins contain predicted disordered regions of 30 residues or longer [11]; and, by analyzing the human disease, it was revealed that many human genetic diseases are caused by alterations of IDPs, that different disease classes vary in the disorder contents of associated proteins, and that many IDPs involved in some diseases are enriched in disorder-based protein interaction sites [33]. In several neurological diseases, the formation of amyloid fibrils and their deposition in various cellular compartments are strictly related to structural disorder and flexibility of IDPs [3,4,5]. Thus, the presence of an amyloidogenic region in proteins is a relevant feature and it has been revealed that more than 80% of human proteins in the disordered protein databases (DisProt + IDEAL) contained one or more amyloid-like portions [34,35]. Experimental computational studies showed that short sequence stretches in proteins may act as nucleating centers for amyloid fibril formation triggering to the aggregation process [36].

**Table 1:** Intrinsically disordered proteins (IDPs) and associated neurodegenerative diseases.

Protein	Diseases
Aβ	Alzheimer’s disease, Dutch hereditary cerebral hemorrhage with amyloidosis, Congophilic angiopathy
Tau	Tauopathies, Alzheimer’s disease, Corticobasal degeneration, Pick’s disease, Progressive supranuclear palsy
Prion protein	Prion diseases, Creutzfeld-Jacob disease, Gerstmann-Strӓussler-Schneiker syndrome, Fatal familial insomnia, Kuru, Bovine spongiform encephalopathy, Scrapie, Chronic wasting disease
α-Synuclein	Synucleinopathies, Parkinson’s disease, Lowy body variant of Alzheimer’s disease, Diffuse Lowy body disease, Dementia with Lowy bodies, Multiple system atrophy, Neurodegeneration with brain iro accumulation type I
β-Synuclein	Parkinson’s disease, Diffuse Lowy body disease
γ-Synuclein	Parkinson’s disease, Diffuse Lowy body disease
Huntingtin’s protein	Huntington’s disease
DRPLA protein	Hereditary dentatorubral-pallidoluysian atrophy
Androgen receptor	Kennedy’s disease or X-link spinal and bulbar muscular atrophy

## 3. DNA Binding Proteins

Disordered regions, terminal tails and flexible linkers are abundant in DNA-binding proteins and play crucial roles in the affinity and specificity to DNA recognizing processes [37]. Also, the interaction with DNA can cause a disorder-to-order transition of IDRs (Intrinsically Disordered Regions) and influence the overall protein–DNA interface dependent on positive charge clustering. Disordered tails may be viewed as DNA recognizing subdomains and favor a “monkey bar” mechanism in which the domains bridge two different DNA fragments simultaneously, where in particular internal disordered linkers can mediate the cross-talks between the domains and their dynamics in an efficient interaction with DNA. A regulation mechanism in DNA recognition is provided by the perturbation of the electrostatic characteristics of the disordered tails by post-translational modifications [37].

**Figure 1:**
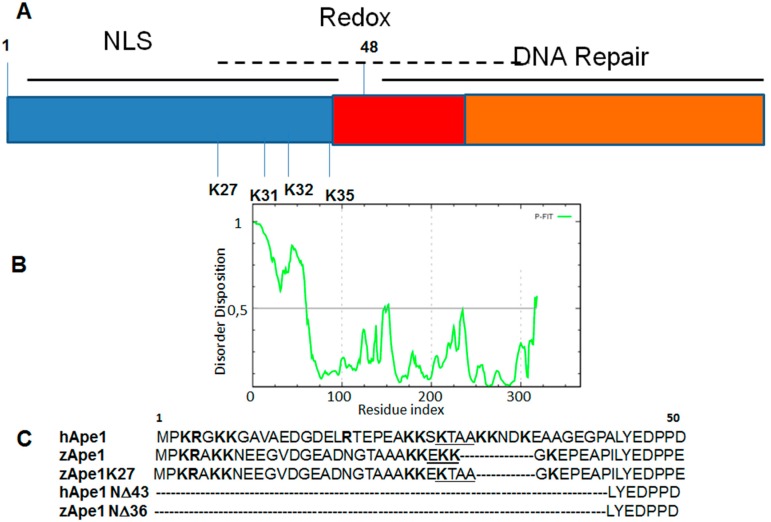
Apurinic/apyrimidinic endonuclease 1 (APE1). (**A**) Schematic representation of its modular structure. NLS: Nuclear Localization Signal; (**B**) Prediction of disorder tendency of hAPE1 sequence with PONDR-FIT; and (**C**) Multiple sequence alignment of the *N*-terminal region of APE1 mutants analyzed in reference [39]; positively charged amino acids are reported in bold while insertional mutations are underlined.

Disordered tails comprise 70% of human DNA-binding proteins and only 50% of non-DNA-binding proteins; moreover the presence of a single tail (both at *N*- or *C*-termini) or two tails is higher in proteins able to bind DNA than in other proteins. The role exerted by the *N*-terminal disordered tail in protein folding/stability and for DNA-interactions was analyzed for homeodomain transcription factors [38] and for several endonucleases [39] both experimentally and computationally. In the absence of DNA, charged residues of the tails may interact with other charged residues and perturb folding as we recently described for Apurinic/apyrimidinic endonuclease 1 (hAPE1) that is the main abasic endonuclease in eukaryotes [39]. This protein is endowed with a modular structure in which the conserved and globular *C*-terminal domain (residues 61–318) is responsible for the enzymatic activity on abasic DNA, while the *N*-terminal portion (residues 1–60) is an unstructured tail mainly devoted to the redox co-activating function toward different transcription factors [40] and to the interaction with other protein partners [41]. In Figure 1A, a schematic representation of APE1 structure and the profile of disorder prediction for the whole human sequence (by using PONDR-FIT [42]) (Figure 1B) are reported. Since the DNA binding activity of hAPE1 is higher in respect to the orthologous protein of phylogenetically distant organisms, such as zebrafish (zAPE1) [41], in our recent study we have analyzed in terms of binding affinities and thermal stabilities several protein variants reported in Figure 1C. Circular dichorism studies, that are generally very useful to investigate oligonucleotides–protein complexes [43,44], provided denaturation temperatures of APE1 variants reported in Table 2 are: by comparing Tm values a strong influence of the *N*-terminal tail on protein stability was pointed out, suggesting a direct correlation between the ability to recognize different oligonucleotides or protein substrates and the flexibility of the *N*-terminal region of APE1 (supported by SPR (Surface Plasmon Resonance) experiments).

**Table 2:** Denaturation temperatures for APE1 mutants.

APE1 Mutants	*T*_m_ (°C)
hAPE1	41.5
zAPE1	46.5
zAPE1 K27	39.5
hAPE1 N∆43	44.0
zAPE1 N∆36	50.5

Furthermore, the high content of charged residues and their non-random distribution along the tail, call for increased understanding of the sequence–structure–function relationship in disordered regions. Perturbing the composition and distribution of charged residues in the disordered regions, by post-translational modifications, may further cause structural variations and influence the capability of the tail to interact nonspecifically with DNA by reducing its DNA affinity. In respect to this, another study on PTM-APE1 [45] revealed that to the nucleolar accumulation of this protein depends on lysine residues in the *N*-tail that undergo acetylation upon genotoxic stress and modulate its BER (base excision repair) activity in cell. These studies pointed out that APE1 *N*-terminal tail represents a flexible device, evolutionary selected to specifically modulate APE1 functions and that can be considered a specific drug-target for the exploration of novel pharmacological strategies, aiming at the functional modulation of the protein using small molecules and/or peptides, as recently carried out through the screening of commercial small molecules libraries [46].

Transcription factors are modular proteins including one or more DNA-binding domains, which recognize and bind to specific sequences of DNA adjacent to the genes that they regulate, and one or more transactivation domains, which are recognized by co-activators and/or other transcription factors and most of them contain foldable IDRs (MoRFs) as, for example, several known complexes of p53 with different partners involve various disordered regions [47]. Studies carried out on these proteins demonstrated that the tail, in the presence of DNA, anchors the protein to DNA through electrostatic forces that modulate and stabilize the folded homeodomain and furthermore the tails may fold upon binding to the DNA in the minor groove or remain partly flexible or disordered [48]. Often the interactions of disordered regions may be realized via multiple segments that assist protein–DNA interactions in various mechanisms and a classic example of this mechanism is represented by *C*-tails of p53 [49].

The p53 protein is a tetrameric transcription factor that plays a key role in cell cycle control: it is a tumor suppressor implicated in induction of cell-cycle arrest, apoptosis, or DNA repair, senescence and differentiation. p53 protein is a homotetramer with independently folded domains that are linked and extended disordered regions that represent about 50% of the protein: an intrinsically disordered *N*-terminal transactivation domain followed by a proline-rich region while the central DNA-binding domain has an immunoglobulin-like β-sandwich fold. The intrinsically disordered proline-rich region plays an important structural role: it serves as a potential site for protein–protein interactions and as a linker region that projects the transactivation domain away from the central DNA-Binding Domain–DNA complex to push out and interact more efficiently with transcriptional coactivators and interestingly contains the most common p53 polymorphism, which has been associated with different cancer risks. The ID *C*-terminal region of p53 is subject to extensive PTMs in both stressed and unstressed cells and displays a unique chameleonic MoRF sequence that can adopt α-helical, β-strand, and coiled conformations upon binding to different regulatory proteins [50]. The large radius of the *C*-tails allows interactions with remote DNA segments enhancing the localization and affinity of p53 to DNA and making the positively charged residues of *C*-tails essential for the modulation of the interactions with DNA. Moreover, the disordered *C*-tails mediate the interactions between the DNA Binding Domain and DNA, and dictate the orientation of the tetramerization domain relative to the DNA, although this orientation is also dependent on the local cellular environment. These results support the fact that the tails promote a conformational change in the p53–DNA complexes [49].

More than two-thirds of eukaryotic proteins are composed of multiple domains and often they lack any interface and can cooperate only via a disordered and flexible linker. Understanding the degree of cooperation between domains tethered by a linker that allows proteins to achieve efficient DNA recognition is essential for understanding cellular network at the molecular level [51]. From the comparisons of the electrostatic contributions to the protein–DNA binding energy made by the individual domains, it has emerged that tethered domains tend to have different DNA-binding affinities suggesting that both specific and nonspecific DNA binding has biological significance. It has been found that although the DNA Binding Domains themselves exhibit significant order, their flanking regions have a significant disorder content, which suggests a functional role for such IDRs in DNA binding. These observations prompted studies focused on testing the roles of flanking regions in determining or modulating the DNA binding affinity and/or specificity. The flexibility of flanking IDRs might contribute to the ability of DNA Binding Domain to (1) appropriately recognize target DNA sequences; (2) bind to a wide diversity of DNA targets; (3) be anchored with high affinity to DNA after recognizing target sequence; (4) bind to other factors and complexes positioned on the DNA or involved in transcriptional regulation or (5) present activation domains to downstream transcriptional regulatory machinery [37].

Among multidomain proteins in which flexible linkers conjunct functional regions, calmodulin (CaM) and nucleophosmin (NPM1) represent interesting examples [52].

CaM is a 148 residue protein with four calcium binding sites that serve to mediate extracellularly induced Ca^2+^ signaling within the cytosol. CaM modulates the activity of a large number of enzymes by direct binding, with both calcium-dependent and calcium-independent binding modes. The regions bound by CaM are typically about 20 residues in length and mostly α-helical in nature but exhibit limited sequence identity and in many cases are non-homologous; thus, a mechanism that incorporates specificity but permits diversity must be encoded in CaM’s structure [53]. The X-ray crystal structure of CaM indicated that it has two homologous globular domains connected by a rigid 26 residue α-helix [54]; then, NMR analyses revealed that residues 77–81 in the middle of this helix were highly flexible and act as a hinge that facilitates a binding mode in which CaM surrounds the target regions of its partners within the two Ca^2+^-binding, globular domains, and in some cases the hinge region remains unstructured after complexes formation [55]. This flexible segment is able to accommodate very different sequences by allowing the CaM surface to find complementary interactions by sampling different positions and orientations. Moreover, it facilitates the separation between the two globular regions upon binding, allowing again for binding diversity [56].

**Figure 2:**
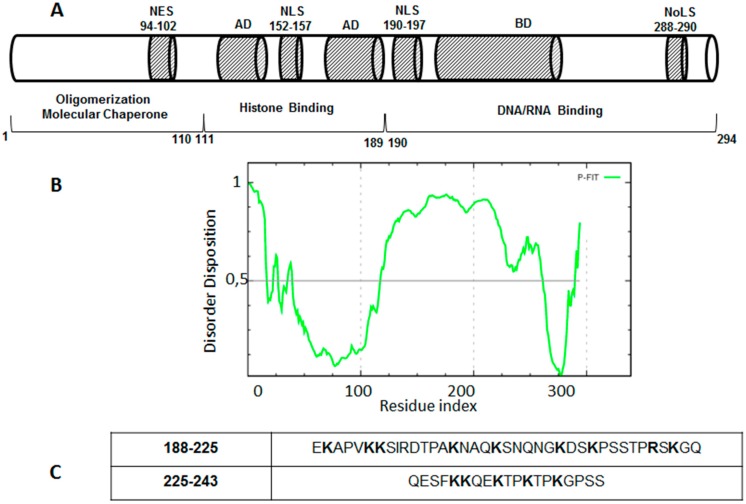
Nucleophosmin 1 (NPM1). (**A**) Schematic representation of its structure. AD: Acidic Domain; BD: Basic Domain; NoLS: Nucleolar Localization Signal; (**B**) Prediction of disorder tendency of NPM1 sequence with PONDR-FIT; and (**C**) Sequences of central intrinsically disordered regions of protein; positively charged amino acids are reported in bold.

NPM1 is an abundant multifunctional protein which is present in high quantities in the granular region of nucleoli [57,58]. It is capable of shuttling between nucleus and cytoplasm [59] and is involved in many cellular functions such as the regulation of ribosome biogenesis, chromatin remodeling, DNA replication, recombination, transcription, repair and the control of centrosome duplication [60,61]. Notably, NPM1 has been identified as the most frequently mutated gene in acute myeloid leukemia (AML) patients, accounting for approximately 30% of cases [62,63,64,65,66]. Besides its primary role as a therapeutic target in AML drug discovery programs, it represents an important model for multidomain proteins [52], and a schematic representation of its modular structure is reported in Figure 2A.

The *N*-terminal domain extends for approximately 100 residues and displays an eight-stranded beta-barrel fold [67,68]. The central portion of NPM1 is characterized by the presence of two acid domains (residues 119–133 and 161–188) and a basic region (residues 198–239) that are intrinsically disordered, as predicted for the whole human sequence (by PONDR-FIT [42]), and recently demonstrated by us and others [69,70], as shown in Figure 2B. Indeed, internal regions have low mean hydrophobicity and high net charge (Figure 2C) as typical of the disordered sequences. The *C*-terminal domain (CTD) forms a globular structure consisting of a three helix bundles and its destabilization abolishes the nucleolar localization of the protein [71]. Despite the structural heterogeneity of the different regions of the protein, many biophysical studies have highlighted their mutual stabilization upon treatments with temperature or chemical denaturants [72,73], and ongoing studies are exploring the role of central intrinsically disordered regions in the cross-talk between folded domains.

Several investigations pointed out that the CTD of NPM1 is crucial to specifically recognize G-quadruplex DNA motifs [74,75,76,77,78,79]. Initial contrasting NMR analyses established that G-quadruplex recognition by NPM1 was primarily due to residues belonging to the helices H1 and H2 of the CTD [75], but more recently molecular dynamics simulations and SPR data indicated that the unstructured region plays a primary role in the mechanism. Besides, facilitating the formation of the DNA-complex through long range electrostatic interactions, it directly contacts the G-quadruplex scaffold through multiple and transient electrostatic interactions significantly enlarging the contact surface [78].

In this scenario, in our recent study, following a rational dissection approach of protein sequence, we unveiled structural and functional determinants of the interaction between G-quadruplex DNA and NPM1. We analyzed the contribution of single protein regions in DNA recognition mechanism through the structural and functional characterization of peptides spanning IDRs and helices of CTD. Our results confirmed that the tight binding of NPM1 to the G-quadruplex is achieved through the cooperation of both folded and unfolded regions that are individually able to bind it [70]. Ongoing studies are focused on the design of peptidomimetics able to stabilize G-quadruplex/NPM1 complex to be tested as potential therapeutics in the field of AML disease.

## 4. Drug Design for the Disordered Proteins

A widely accepted methodology for design drugs targeting IDPs is not yet available. Although there are several successful analyses, the flexibility of the dynamic structure must be fully considered to design a molecule against IDRs [80]. In many cases, IDPs undergo a conformational transition during a binding event [36] and molecular dynamics (MD) simulations studies can help to unveil the mechanism [81]. During MD simulation, equilibrium fluctuations of proteins are explored and cryptic sites, formed only upon the binding, are investigated [82,83]. However, a great challenge is the knowledge of time-scale parameters that properly represent the involved equilibria.

NMR remains the best technique to experimentally observe and characterize the structural dynamics of IDRs. ^13^C detection provides a valuable tool as ^13^C nuclei are characterized by a good chemical shift dispersion even in absence of a stable 3D structure not affected by hydrogen-exchange-induced line broadening [84].

Drug discovery processes for IDPs can be divided into two main categories: (1) studies focused on drug-like small molecules able to interfere with the aggregation process of intrinsically disordered proteins and to stabilize their soluble monomeric form in order to influence downstream aggregation events, including the formation of oligomeric species that are the origin of neuronal damage; and (2) investigations aimed to identify modulators of PPIs involving IDPs.

So far, only a few examples of short peptides and small molecules against IDPs are available [47,85,86,87] and are summarized in Table 3, in which the nature of the inhibitor and employed structural technique is outlined. A recent strategy combining dynamic simulations and fragment based drug design identified small molecules able to bind to Aβ42 peptide [88]. The approach was aimed at introducing flexibility in docking and at the identification of a series of highly populated clusters of conformations within the Aβ42 structural ensemble. A library of small-molecule fragments was screened *in silico* to find molecules able to bind to specific “hot spot” regions in a given conformation of a protein. Several natural drug-like molecules demonstrated their ability to inhibit oligomerization/fibrillation processes: the polyphenol-(−)-epigallocatechin gallate (EGCG) showed antifibril activity against a variety of targets [89] as well as pthalocyanine tetrasulfate (PcTS) toward τ-protein [90]. Also, the antibiotic rifamycin SV revealed a fibrillar inhibitor of β2-microglobulin able to bind to unfolded protein monomers and to shift them toward no amyloid-like aggregates [91], and the peptide carnosine demonstrated an ability to inhibit amyloid growth via the perturbation of the hydrogen-bond network near residues that play some key roles in Aβ fibrillation [92].

On the other hand, because of their involvement in the pathogenesis of various human diseases, PPIs related to IDPs represent novel and attractive targets. Below, reported data (Table 3) show that the selective blockade of specific interactions of IDPs with their binding partners is possible [47] and, similarly to structured PPIs, it is crucial to decode their hot spots. Such hot spots often can be localized as hydrophobic clusters in helix-forming molecular recognition elements and mimicking these hydrophobic clusters could block interaction. Computational tools have been developed to locate such druggable short disordered binding regions which fold upon binding into a specific structural element [93,94].

A generalized method to inhibit complexes where recognition of an IDPR mediates a crucial interaction was provided by Cheng *et al.* [93]. It predicts important IDPR recognition sequences and employs these regions as starting compounds to find interactions and design mimetics. The system uses bioinformatics: this method can allow for access to pathways and interactions that, unlike p53–MDM2, are not fully characterized structurally or even fully mapped, but as proof of concept, it was applied to the p53–MDM2 complex.

The interaction of p53 with MDM2 protein prevents tumor suppressor activity and targets p53 for ubiquitination and degradation. The binding site on p53 is localized to residues which are intrinsically disordered and fold into a α-helix to bind in a groove on MDM2. Structure-based drug design was employed to guide discovery of both peptidomimetics and small molecules (nutlins) able to bind to the MDM2 groove and displace the p53 helix, releasing it back to its disordered state [95,96].

Homo- and hetero-dimerization processes involving newly formed helices are often at the basis of PPIs involving IDPs and have been the target of many structure-based drug designs. For example, it associated with the activation of Kaposi’s sarcoma associated herpes virus protease (KSHV Pr) [97] activity and the dimer interface has been selected in a search for inhibitors. As with p53, a helix mimetic strategy led to an initial hit chemically modified to obtain the small molecule DD2 able to bind to structured surface residues of the protein to stabilize the monomeric form [98]. Another case is represented by the hetero-dimer between c-Myc/Max proteins both belonging to HLHZip (Helix-Loop-Helix-Zipper) family of transcription factors that is dysregulated in the majority of human cancers. Both a peptidomimetic combinatorial and a “credit-card” library with a naphthyl core able to mime the largely flat and hydrophobic interface of PPI yielded to inhibitors [99,100,101,102].

**Table 3:** Summary of several drug-discovery studies for the identification of lead compounds against IDPs.

Protein/Complex	Inhibitor	Structural Technique	Reference
Aβ42	Curcumin, Congo red	*In silico* FBDD	[86]
Aβ42, αsynuclein, IAPP	EGCG	CD, NMR	[87]
Aβ42	carnosine	NMR	[90]
τ-protein	PcTS	SAXS, NMR, EPR	[88]
β2 microglobulin	rifamycin SV	ESI-IMS-MS	[89]
p53/MDM2	peptidomimetics, small molecules	Virtual screening	[93]
KSHV Pr	small molecule	NMR	[96]
c-Myc/Max	peptidomimetic, small molecule	Virtual screening, FRET, NMR, FP	[97,98,99,100]
c-Fos/c-Jun	peptidomimetic, small molecule	MD, FP	[101,102]
androgen receptor	peptidomimetic	X-ray	[103]

FBDD: Fragment-Based Drug Design; CD: Circular Dichroism; NMR: Nuclear Magnetic Resonance; SAXS: Small-Angle X-ray Scattering; EPR: Electron Paramagnetic Resonance; ESI-IMS-MS: Electrospray Ionization-Ion Mobility Spectrometry-Mass Spectrometry; FRET: Forster Resonance Energy Transfer; FP: Fluorescence Polarization.

Also, the basic regions of the c-Fos/c-Jun hetero-dimer were addressed by using a cyclic peptide inhibitor as a starting compound. Successively, the development of a pharmacophore model led to identifying several small molecules capable of inhibiting coupled binding and folding of the basic region [103]. A HTS study for inhibitors of ΔFosB DNA binding provided two inhibitors, one of them that did not perturb the degree of helicity in the protein [104].

Naturally extracted chlorinated peptide sintokamides from marine sponge (*Dysidea* sp.) were analyzed for the inhibition of androgen receptor in an irrational screening approach. Although the specific binding site was not identified, sintokamide A resulted in the inhibition of transcriptional control [105].

## 5. Conclusions

The main IDPRs are highly dynamic even if their structures can be described as a limited number of lower-energy conformations [106]. The structural adaptability of IDPs provide unique capabilities for them to interact with multiple protein partners without losing specificity, in contrast to ordered proteins with well-defined architecture that interact mainly with a single protein partner. Structural biology and MD simulations can help to address questions related to what is the structural basis for promiscuous binding and the mechanisms that lead to specific responses to a particular cellular signal [107].

Usually, therapeutic compounds accomplish their regulator function by binding to small cavities/grooves within target proteins, and the unfolded nature of IDPs makes drug design difficult owing to severe limitations in this methodology [108]. However, the frequent occurrence of intrinsic disorder in disease-associated proteins strongly suggests that disorder information has to be deeply evaluated in the drug discovery process towards the development of novel therapeutic compounds. Unfortunately, this area remained largely unexplored primarily due to the lack of effective screening tools. Although it is very challenging to design small molecules for such targets that constantly change their overall architecture, successful studies are reported. From recent investigations it is emerging that the crucial step to make IDPs “druggable” targets is the identification of “hot spots” involved in complex formation and natural molecules such as interacting protein regions (peptides or peptidomimetics) that result from nature’s combinatorial chemistry. The resultant chemical diversity and ability to interact with multiple biological target molecules might be good starting points for drug discovery platforms [109].
